# Placebo by Proxy in Neonatal Randomized Controlled Trials: Does It Matter?

**DOI:** 10.3390/children4060043

**Published:** 2017-05-30

**Authors:** Tiziana L. Burkart, Andrea Kraus, Brigitte Koller, Giancarlo Natalucci, Beatrice Latal, Jean-Claude Fauchère, Hans Ulrich Bucher, Christoph M. Rüegger, for the Swiss EPO Neuroprotection Trial

**Affiliations:** 1 Department of Neonatology, University Hospital Zürich, 8091 Zürich, Switzerland; tiziana.burkart@gmail.com (T.L.B.); BrigitteMaria.Koller@usz.ch (B.K.); Giancarlo.Natalucci@usz.ch (G.N.); Jean-Claude.Fauchere@usz.ch (J.-C.F.); buh@usz.ch (H.U.B.); 2 Department of Mathematics and Statistics, Masaryk University, 611 37 Brno, Czech Republic; andrea.kraus@mail.muni.cz; 3 Child Development Centre, University Children’s Hospital, 8032 Zürich, Switzerland; bea.latal@kispi.uzh.ch; 4 Swiss EPO Neuroprotection Trial Group (members listed in the *Acknowledgements* section)

**Keywords:** preterm infants, placebo by proxy, long-term outcome, randomized controlled trial

## Abstract

Placebo effects emerging from the expectations of relatives, also known as placebo by proxy, have seldom been explored. The aim of this study was to investigate whether in a randomized controlled trial (RCT) there is a clinically relevant difference in long-term outcome between very preterm infants whose parents assume that verum (PAV) had been administered and very preterm infants whose parents assume that placebo (PAP) had been administered. The difference between the PAV and PAP infants with respect to the primary outcome–IQ at 5 years of age–was considered clinically irrelevant if the confidence interval (CI) for the mean difference resided within our pre-specified ±5-point equivalence margins. When adjusted for the effects of verum/placebo, socioeconomic status (SES), head circumference and sepsis, the CI was [−3.04, 5.67] points in favor of the PAV group. Consequently, our study did not show equivalence between the PAV and PAP groups, with respect to the pre-specified margins of equivalence. Therefore, our findings suggest that there is a small, but clinically irrelevant degree to which a preterm infant’s response to therapy is affected by its parents’ expectations, however, additional large-scale studies are needed to confirm this conjecture.

## 1. Introduction

Traditionally, the placebo effect is considered the response of a reference group following the administration of an inert substance that needs to be controlled for in clinical trials [1]. However, the emerging field of placebo studies is producing scientific evidence that these more intangible elements of treatment could also essentially improve patient outcomes [2,3]. In this regard, the concept of placebo by proxy has emerged, suggesting that the beliefs and expectations of family members and healthcare providers might also affect the patient’s response to therapy [4]. Although considerable research has been devoted to the placebo by proxy effect in veterinary medicine, where caregiver placebo effects seem to be common [5], less attention has been paid to its structured investigation in humans, particularly in a pediatric population, where this effect would also be expected. Whalley and Hyland investigated whether the efficacy of an impure placebo (Bach flower therapy, a homeopathic remedy) used to improve the symptoms of temper tantrums in 2- to 5-year-old children was affected by the parents’ beliefs and moods [6]. The authors found a sustained and significant improvement in tantrum frequency and severity, which was strongly correlated to the parents’ beliefs of the treatment given.

Preterm infants and their families could offer an ideal opportunity for investigating placebo by proxy effects. Intrinsic placebo effects in newborns are believed to be non-existent, due to their limited ability to understand the significance of a therapy. However, within a randomized controlled trial (RCT) targeted at improving long-term neurodevelopment outcome, placebo by proxy effects originating from their parents may be conceivable, due to the parent`s significant and long-lasting impact on their infants. Therefore, we aimed to investigate whether the long-term outcome of very preterm infants within an RCT is influenced by parental beliefs and expectations regarding their treatment allocation. We hypothesized that the infants’ outcomes would be unaffected by placebo by proxy when assessed at preschool age.

## 2. Materials and Methods

Our study group consisted of very preterm infants born between 26 0/7 and 31 6/7 gestational weeks enrolled in an RCT lasting from 2005 to 2012 (www.clinicaltrials.gov; NCT00413946) [7]. The primary objective of that RCT was to investigate the neuroprotective effect of early high-dose recombinant human erythropoietin (rhEPO) in very preterm infants, with the goal of improving their long-term neurodevelopmental outcome. Infants were excluded for any of the following reasons: genetically defined syndromes, severe congenital malformations adversely affecting life expectancy or neurodevelopment, and infants who were admitted for palliative care a priori. Within the first three hours of life, the infants were randomized to receive rhEPO (3000 IU per kilogram body weight dissolved in 1 mL distilled water, maximal dose was 4500 IU) or placebo (1 mL NaCl 0.9% per kilo body weight, maximal dose 1.5 mL) intravenously at 3, 12–18, and 36–42 h after birth. Other than the study treatment, all procedures and examinations were standard practice of care. As part of the trial’s secondary outcomes, the intelligence quotient was assessed at the follow-up examination at the age of 5 years (FU5) using the Kaufmann Assessment Battery for Children (K-ABC) [8]. 

Of all the infants enrolled in the EPO trial, only those who had been examined with the K-ABC by the end of the year 2015 were considered in the present placebo by proxy study. The 2015 cut-off was chosen to ensure that 50% of the EPO trial participants would be available for further analysis. The infants with incomplete follow-up data at FU5 were excluded. Data on the included infants were entered into a database maintained by the Swiss Neonatal Network & Follow-up Group. Socioeconomic status (SES) was determined by a 12-point scale that is based on paternal occupation and maternal education (each with a score of 1–6; total score 2–12; a low score indicated high income/education) [9]. Pregnancy complications were defined as either the presence of chorioamnionitis, gestational diabetes, preeclampsia, or hemolysis, elevated liver enzymes, low platelet count (HELLP) syndrome.

At FU5, all parents were given a questionnaire to express their personal feeling on the group allocation of their infants—either treatment or placebo group. The two groups, infants whose parents assumed that verum (PAV) had been administered and infants whose parents assumed that placebo (PAP) had been administered, were finally compared with regard to our variable of interest, the IQ at 5 years of age.

The EPO trial was approved by the Committee of the University Children’s Hospital Zurich, by the Ethical Committee of the Canton Zurich, and by SwissMedics Berne. Written informed consent was obtained from all parents.

### 2.1. Statistics

Baseline characteristics and short-term outcomes were compared using the Mann-Whitney test for continuous variables and Fisher’s exact test for nominal variables. Equivalence of the two groups with respect to the primary outcome was assessed by an equivalence test [10,11]. Standard situations aim to show that the outcome is different in different groups of interest (and where so-called superiority testing is used). In contrast, our aim was to show that the between-group difference in the outcome does not exceed a pre-specified margin of clinical relevance, hence the use of equivalence testing. To perform an equivalence test at the level of 5%, a 90% confidence interval (CI) is constructed for the mean difference between the outcomes in the two groups. Equivalence is shown if the whole interval is contained between −Δ and Δ. We set the margin of clinical relevance at Δ = 5 points (0.3 standard deviations (*SD*) of the K-ABC).

We constructed unadjusted CIs based on the *t*-test, as well as CIs based on linear regression, where we adjusted for characteristics that were either not well-balanced at baseline or had a significant impact on the IQ at 5 years of age. As the neuroprotective effect of EPO is currently under investigation [7], we had decided prior to the analysis to also adjust for the effect of verum/placebo.

## 3. Results

### 3.1. Study Population

A total of 243 randomized infants, with a 5-year follow-up examination by the end of 2015, were available for this study. Of those, 108 infants with completed questionnaires were included in our analysis: 57 had been randomized to the EPO and 51 to the placebo group. Besides pregnancy complications (*p* < 0.01), sex (*p* < 0.01), and SES (*p* = 0.02), no significant baseline difference was observed between included and not included infants (Table 1).

**Figure 1:**
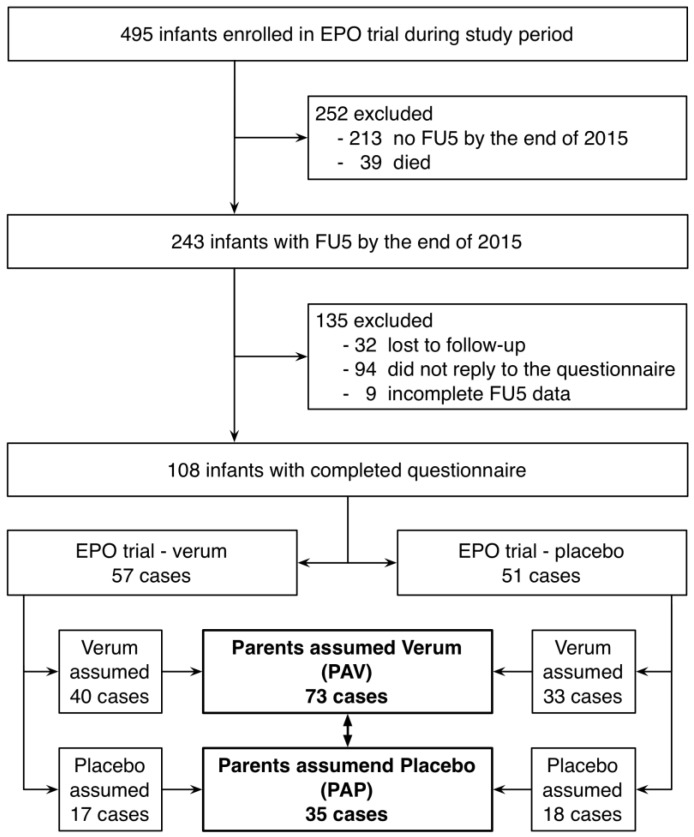
Flow chart of the sample size. FU5 = 5 years of age.

Although SES was a significant predictor of the IQ at 5 years of age, the IQ difference between included and not included infants was not significant (*p* = 0.07). The parents of the included infants believed that verum had been administered in 73 (68%) cases and placebo in 35 (32%) cases (Figure 1).

At baseline, the perinatal variables were evenly distributed between the PAV and the PAP group (Table 2). At the time of discharge home, neonatal morbidity rates were slightly higher in PAV (all parents of infants suffering from major intraventricular hemorrhage, sepsis, and necrotizing enterocolitis believed that verum had been administered to their infants) than in PAP group, but the difference reached a statistically significant level only for sepsis (*p* = 0.03).

### 3.2. Comparison Between Groups

The mean IQ at FU5 was 98.4 points in the PAV and 98.4 points in the PAP group. The unadjusted 90% CI for the difference in mean IQ was from −4.23 to 4.24 points (PAV-PAP). As explained in the methods section, we adjusted the CI for the effect of verum/placebo. Among all baseline characteristics, SES (*p* < 0.01), birth weight (*p* = 0.02) and head circumference at birth (*p* = 0.02) were significantly associated with the IQ at FU5, birth weight being strongly correlated with head circumference (Pearson correlation coefficient 0.88, *p* < 0.01). Therefore, although these factors did not appear to be imbalanced between the two groups, we adjusted the CI for the mean difference in the IQ between the PAV and PAP groups for SES and head circumference at birth. Although not significantly associated with the IQ at FU5, we adjusted the CI for sepsis as well, because sepsis was not evenly distributed between the PAV and PAP groups. 

**Figure 2:**
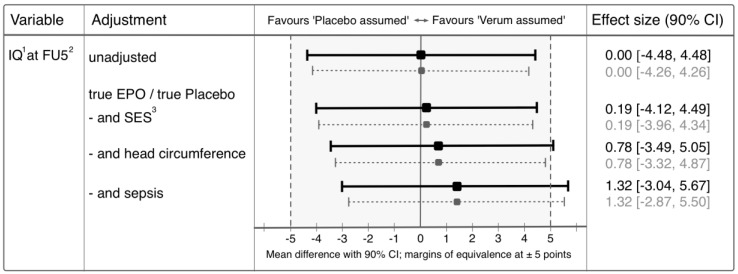
Confidence intervals (CIs) for the difference in mean IQ at FU5 between the PAV and PAP groups: unadjusted and gradually adjusted for the effect of verum/placebo and SES, head circumference, and sepsis. CIs in solid black lines are based on a linear regression using data of infants whose parents answered the questionnaire and had complete information on verum/placebo, SES, head circumference, and sepsis (*n* = 105); CIs in grey dotted lines are based on a bias-corrected missing indicator method, using data of all infants with complete information on verum/placebo, SES, head circumference, and sepsis (infants of parents with (*n* = 105) and without (*n* = 83) completed questionnaire). ^1^ IQ: intelligence quotient, ^2^ FU5: 5-year follow-up examination, ^3^ SES: socioeconomic status.

After adjustment for the effect of verum/placebo, SES, head circumference, and sepsis, the CI for the difference in mean IQ at FU5 between the PAV and PAP groups was [−3.04, 5.67] points (PAV-PAP, Figure 2, solid black CIs).

## 4. Discussion

With respect to our pre-specified definition of equivalence, the outcome of infants enrolled in an RCT and allegedly treated with verum was not shown to be equivalent to the outcome of infants allegedly treated with placebo. After adjusting for variables that might have influenced the outcome or that were imbalanced between the PAV and PAP groups, the CI for the mean difference in IQ (PAV-PAP) crossed the upper equivalence margin by 0.67 points.

Supposing the existence of placebo by proxy in neonatal care, knowledge about such elusive interactions among patients, their families, and their health care providers is important given that they might impact clinical practice in various ways. First, placebo by proxy might influence clinical decision-making. Several studies have examined how parental expectations and/or physician perceptions of those expectations affect the physician’s treatment rationale and management plan [12,13]. Enabling parents to be central to the care process of their infants, to take part in rounds, and to collaborate on their infant’s care plan, as proposed in the family integrated care model [14], might further increase such reciprocal effects. Second, placebo by proxy could influence the estimations of treatment outcomes in trials and should in this context be considered as an integral part of the so-called trial effect [15]. It must be assumed that study endpoints favoring the perceptions of clinicians or family members over objective markers of the patient response are especially prone to placebo by proxy bias. This may partially explain a larger placebo response in children than in adults participating in clinical trials [16,17].

Not yet fully understood are the mechanisms that might underlie placebo by proxy in our set of preterm infants, even though the neurophysiological pathways behind classical placebo effects in adult patients are well characterized [18,19]. One potential mechanism involves the conscious expectancy of the parents; the content of information related to the treatment, either positive or negative, can manipulate parental expectations and mediate placebo effects [20]. Closely related is a second mechanism called associative learning, which is derived from previous experiences of positive or negative therapeutic outcomes. It has been shown that observational social learning produced placebo responses that were similar to those induced by directly experiencing the benefit [21]. A third mechanism finally refers to the fact that premature birth is recognized as a stressful and emotionally demanding experience that has a long-term impact on both parents [22]. Previous research shows that the mothers of preterm infants are well recognized as being more controlling, intrusive, and active in their interactions with their infant [23]. The sicker the infant and the more pronounced the maternal posttraumatic stress symptoms are, the stronger such behavioral changes and thus potential placebo by proxy effects seem to be [24,25]. The results of our study may be of support for this theory, given that the infants in the PAV group showed a slightly increased incidence of neonatal morbidities than those in the PAP group (Table 2). Even if these differences were weak and reached statistical significance only for sepsis, they might nevertheless reflect the strong parental hopes and wishes for a potentially effective therapy when faced with a sick preterm infant. Therefore, it was found that all three mechanisms did not in fact have a genuine change in the newborn’s condition, but instead, an altered behavior of parents and caregivers was revealed, which may result in higher encouragement, attention, and support towards their infants and consequently, in a modified psychosocial context of the newborn. Such characteristic parent–child interactions suggest that placebo by proxy effects might exist in neonatal conditions. This is in line with the results of a recent meta-analysis about the placebo response in patients with a limited ability to understand the significance of a therapy [26]. The authors showed that these patients improve in the placebo arm of an RCT and found a significant effect of age, indicating higher placebo responses explained by stronger placebo by proxy effects in the treatment of younger patients.

There are several limitations to the findings presented in this report. (1) An observational study design was used, because other methods such as randomization could not be applied (parental perception of allocation cannot be assigned). (2) The ±5-point equivalence margins mark the range of differences in the IQ that we consider clinically irrelevant. Our adjusted IQ difference was estimated at 1.32 points, which is well within the equivalence margins. However, because the IQ difference is only estimated, uncertainty (expressed here as the CI) needed to be considered as well. With our sample size, the uncertainty was ±4.35 points. This is so close to ±5 points that we could only have shown equivalence had the estimated difference been within ±0.65 points. Hence, with the IQ difference of 1.32 points, we did not show equivalence. However, we believe that with a larger study, we would obtain a shorter CI (smaller uncertainty) and thus show equivalence. (3) Missing data was a limitation in this study. Our questionnaire unreturned rate was 47%, which is consistent with this format of data collection (postal survey) [27]. Unfortunately, missing data introduces a possibility of bias and also reduced our sample size by almost half. To address the first concern, we compared the 108 infants with a completed questionnaire to the 94 infants without a completed questionnaire: the two groups did not differ significantly in their IQ at FU5 (*p* = 0.07), nor did the effects of verum/placebo, SES, head circumference, and sepsis on the IQ appear different in the two groups (*p* = 0.33). To address the loss in sample size (leading to a wider CI), we used the available information of 83 infants without a completed questionnaire but with complete information on IQ, verum/placebo, SES, head circumference, and sepsis to obtain more precise estimates of the effects of verum/placebo, SES, head circumference, and sepsis on the IQ. This allowed us to construct a shorter CI for the mean difference in IQ between the PAV and PAP groups adjusted for those effects (we used a bias-corrected missing indicator method [28]). The resulting CI was [−2.87, 5.50] points (Figure 2, dotted grey CIs), which supports our conjecture that we might be successful in showing equivalence within a larger study.

## 5. Conclusions

Our research on placebo by proxy in very preterm infants did not find an equivalence in the IQ at five years of age between infants whose parents believed that verum had been administered and infants with parents who believed that a placebo had been administered. Parental beliefs and expectations may have a measurable impact on their preterm infant’s response to therapy at preschool age; however, this impact appears to be small and clinically irrelevant, and needs to be confirmed within a larger study.

## Figures and Tables

**Table 1:** Baseline characteristics and outcomes of all infants with FU5 by the end of 2015.

Characteristics	Included in the Present Study (*n* = 108)	Not Included in the Present Study (*n* = 135)	*p*-value ^b^
Pregnancy complications ^1^	48/108 (44.4)	89/135 (65.9)	<0.01
Prenatal steroids	103/108 (95.4)	129/135 (95.6)	1.00
Male sex	73/108 (67.6)	67/135 (49.6)	<0.01
Gestational age (weeks)	28.9 ± 1.8	29.2 ± 1.7	0.14
Birth weight (g)	1177 ± 322	1183 ± 351	0.95
Head circumference at birth (cm)	26.7 ± 2.2	26.6 ± 2.1	0.58
Intraventricular hemorrhage III–IV	6/108 (5.6)	3/134 (2.2)	0.19
Sepsis	9/108 (8.3)	16/135 (11.8)	0.40
Retinopathy of prematurity (any stage)	9/107 (8.4)	14/134 (10.4)	0.66
Necrotizing enterocolitis	4/108 (3.7)	3/135 (2.2)	0.70
Bronchopulmonary dysplasia	35/108 (32.4)	45/135 (33.3)	0.89
Maternal age, years	32.1 ± 5.5	31.9 ± 5.9	0.70
SES	5.6 ± 2.3	6.2 ± 2.4	0.02

Values are the means ± *SD* or *n* (%). FU5 = 5 years of age; ^1^ Chorioamnionitis, gestational diabetes, preeclampsia, or HELLP (hemolysis, elevated liver enzymes, low platelet count) syndrome; ^b^ Mann-Whitney or Fisher’s test; SES = socioeconomic status.

**Table 2:** Perinatal, neonatal, and sociodemographic baseline characteristics of infants included within the study.

Characteristics	Parents Assumed Verum (PAV) (*n* = 73)	Parents Assumed Placebo (PAP) (*n* = 35)	*p*-value ^b^
Pregnancy complications ^1^	34/73 (46.6)	14/35 (40.0)	0.54
Prenatal steroids	69/73 (94.5)	34/35 (97.2)	1.00
Male sex	49/73 (67.1)	24/35 (68.6)	1.00
Gestational age (weeks)	28.7 ± 1.8	29.2 ± 1.6	0.24
Birth weight (g)	1166 ± 336	1198 ± 296	0.69
Head circumference at birth (cm)	26.5 ± 2.3	27.0 ± 1.9	0.23
Intraventricular hemorrhage III–IV	6/67 (8.2)	0/35 (0.0)	0.17
Sepsis	9/73 (12.3)	0/35 (0.0)	0.03
Retinopathy of prematurity (any stage)	7/73 (9.6)	2/34 (5.9)	0.72
Necrotizing enterocolitis	4/73 (5.5)	0/35 (0.0)	0.30
Bronchopulmonary dysplasia	25/73 (34.2)	10/35 (28.6)	0.66
SES	5.6 ± 2.2	5.5 ± 2.4	0.60
Maternal age (years)	32.3 ± 5.6	31.6 ± 5.4	0.40

Values are the means ± *SD* or *n* (%). ^1^ Chorioamnionitis, gestational diabetes, preeclampsia, or HELLP (hemolysis, elevated liver enzymes, low platelet count) syndrome; ^b^ Mann-Whitney or Fisher’s test.
